# Exploring Perturbations in Peripheral B Cell Memory Subpopulations Early after Kidney Transplantation Using Unsupervised Machine Learning

**DOI:** 10.3390/jcm12196331

**Published:** 2023-10-01

**Authors:** Ariadni Fouza, Anneta Tagkouta, Maria Daoudaki, Maria Stangou, Asimina Fylaktou, Konstantinos Bougioukas, Aliki Xochelli, Lampros Vagiotas, Efstratios Kasimatis, Vasiliki Nikolaidou, Lemonia Skoura, Aikaterini Papagianni, Nikolaos Antoniadis, Georgios Tsoulfas

**Affiliations:** 1 Department of Transplant Surgery, Medical School, Aristotle University of Thessaloniki, General Hospital “Hippokratio”, 54642 Thessaloniki, Greece; lampisv@yahoo.gr (L.V.); nikanton@auth.gr (N.A.); tsoulfas@auth.gr (G.T.); 2 Laboratory of Biological Chemistry, Medical School, University Campus, Aristotle University of Thessaloniki, 54124 Thessaloniki, Greece; taganneta@hotmail.com; 3 Department of Hygiene, Social-Preventive Medicine & Medical Statistics, Medical School, University Campus, Aristotle University of Thessaloniki, 54124 Thessaloniki, Greece; mpougioukas@auth.gr; 4 1st Department of Nephrology, Medical School, Aristotle University of Thessaloniki, Hippokration General Hospital, 54642 Thessaloniki, Greece; mstangou@auth.gr (M.S.); frasci@outlook.com.gr (E.K.); aapapagi@auth.gr (A.P.); 5 Department of Immunology, National Peripheral Histocompatibility Center, Hippokration General Hospital of Thessaloniki, 54642 Thessaloniki, Greece; fylaktoumina@gmail.com (A.F.); aliki.xochelli@gmail.com (A.X.); basoniko@hotmail.com (V.N.); 6 Department of Microbiology, Medical School, Aristotle University of Thessaloniki, AHEPA Hospital, 54124 Thessaloniki, Greece; lemskour@auth.gr

**Keywords:** B lymphocytes, B cell subsets, kidney transplantation

## Abstract

Background: B cells have a significant role in transplantation. We examined the distribution of memory subpopulations (MBCs) and naïve B cell (NBCs) phenotypes in patients soon after kidney transplantation. Unsupervised machine learning cluster analysis is used to determine the association between the cellular phenotypes and renal function. Methods: MBC subpopulations and NBCs from 47 stable renal transplant recipients were characterized by flow cytometry just before (T0) and 6 months after (T6) transplantation. T0 and T6 measurements were compared, and clusters of patients with similar cellular phenotypic profiles at T6 were identified. Two clusters, clusters 1 and 2, were formed, and the glomerular filtration rate was estimated (eGFR) for these clusters. Results: A significant increase in NBC frequency was observed between T0 and T6, with no statistically significant differences in the MBC subpopulations. Cluster 1 was characterized by a predominance of the NBC phenotype with a lower frequency of MBCs, whereas cluster 2 was characterized by a high frequency of MBCs and a lower frequency of NBCs. With regard to eGFR, cluster 1 showed a higher value compared to cluster 2. Conclusions: Transplanted kidney patients can be stratified into clusters based on the combination of heterogeneity of MBC phenotype, NBCs and eGFR using unsupervised machine learning.

## 1. Introduction

Transplantation is the most effective treatment for patients with end-stage renal disease (ESRD).

Studies of the alloreactive humoral response have focused on the role of B cells in the generation of donor-specific antibodies (DSAs) and their contribution to mediated rejection, a major cause of allograft loss in renal transplantation.

In addition to their role in generating antibody responses, B cells have antibody-independent functions, including antigen presentation, modulation of T cell differentiation and production of regulatory and pro-inflammatory cytokines. A few years ago, the importance of B regulatory cells in the development of tolerance to kidney transplants came to light [1,2].

Information on the circulating memory B lymphocyte pattern at different time points after transplantation and its contribution to alloreactive responses in kidney transplantation is limited [3,4].

Flow cytometry and the combined detection of CD19 and CD27 markers were used to differentiate between CD19 + CD27^_^ naïve B cells (NBCs), antigen-naïve cells, and CD19 + CD27+ memory B cells (MBCs) [5,6]. MBCs can be further analysed via co-staining with IgD in the following well-defined sub-populations in the blood [7,8]: class-switched (S), IgD-CD27 + , MBCs, class non-switched (NS), IgD + CD27+ MBCs and double negative (DN) IgD− CD27 − MBCs [9,10].

For the peripheral equivalent of marginal zone B cells (MZBCs), a CD19 + CD27+ IgD + IgM+, anti-IgM-labelled monoclonal antibody was also used. MZBCs are innate-like cells, respond rapidly to pattern recognition receptors such as TLR4 [11] and are associated with kidney rejection [12] and prolongation and survival of cardiac allografts upon their depletion. The innate like nature of the cells has been demonstrated in rejected heart transplants [13], where infiltrating B cells respond to innate stimuli [14,15].

Anticipated changes in memory B cell subpopulations early after transplantation, their possible role in graft function outcome and their value as potential biomarkers in renal transplantation are still under investigation.

The aim of the present study is to prospectively evaluate changes to naïve and memory B lymphocyte subpopulations early after transplantation. To investigate the detailed distribution of naïve and MBC subpopulation phenotypes in kidney recipients, a clustering technique will be used to identify distinct immunotypes in relation to graft function to create a kidney recipient profile.

This will be used in the clinical follow-up of the recipients to assess whether it is a reliable predictor of transplant outcome.

## 2. Materials and Methods

### 2.1. Study Patients

Patients undergoing kidney transplantation (KT) were evaluated at time of transplantation and prospectively followed for a period of 6 months.

**Inclusion criteria:**

Patients enrolled in the study were adults, aged 18–60 years, who had been followed at the chronic kidney disease outpatient clinic of the nephrology department for at least two years prior to transplantation.

All subjects in the study were vaccinated in accordance with national vaccination schedules and were included in the study two months or more after the vaccination.

**Exclusion criteria:**

Patients were excluded from the study if they had a history (more than 5 years) of malignancy, autoimmune disease, haematological disease, or treatment with monoclonal antibodies against B or T lymphocytes; if they had a recent (less than 3 months) cytomegalovirus or bacterial infection; if they had an acute deterioration in renal function of unknown cause and/or with a follow-up of less than 2 years. Recipients from deceased donors with cardiac death were also excluded, as were patients who did not comply with treatment instructions.

### 2.2. Study Schedule

Each patient who received KT was eligible based on the inclusion criteria described above.

The day of KT was considered the day of enrolment and defined as T0. Demographic and clinical data, as well as data on medical history, primary disease and treatment, were obtained from patient records. Blood samples were taken before KT and before any immunosuppressive treatment and processed for laboratory and immunological evaluation. After KT, patients were followed up with in the KT outpatient clinic and their renal function, medications and possible side effects were recorded every month. Renal function was based on the estimated glomerular filtration rate (e-GFR) calculated via the 2009 CKD-EPI (Chronic Kidney Disease Epidemiology Collaboration) formula [16].

Fifty-four (54) patients were initially recruited, of whom seven dropped out during follow-up: two due to age over 65 years, three because of relapse of the protopathic disease, one because of very low lymphocyte counts and low B lymphocytes (CD19 + <500) not due to rejection and one with rejection, leaving forty-seven (47) patients to complete the study.

Their immune profile, including naïve and B cell memory subpopulations, was assessed at two time points, T0 and after 6 months of follow-up (T6), so that comparisons pre- and post-transplant could take place.

**Figure 1:**
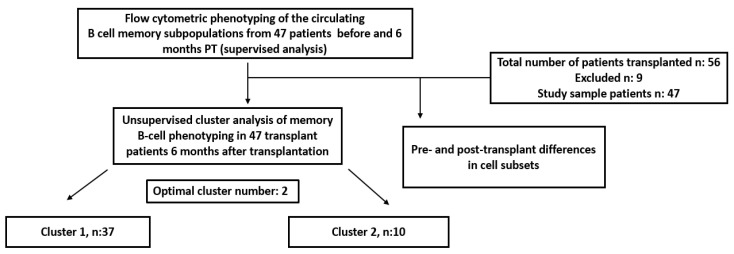
Flow chart of the study design.

Cluster analysis of the immunophenotypic profile of the patients created clusters with similar patient profiles at T6, Figure 1.

### 2.3. Ethics Approval and Consent to Participate

The study was conducted in accordance with ethical regulations and the principles of the Declaration of Helsinki and with the approval of the Institutional Review Board of the Medical School at Aristotle University of Thessaloniki (ref. no. 4356/26-1-2021). Written informed consent was obtained from each patient.

### 2.4. Immunosuppresion Regimen

All patients received the same immunosuppressive regimen, according to the Immunosuppressive Protocol, including steroids, tacrolimus, mycophenolate mofetil and basiliximab or antithymocyte globulin.

Basiliximab was used as induction immunosuppression in 84.5% of patients.

### 2.5. Flow Cytometry-B Cell Phenotyping

Phenotypes of immune cell subsets were defined according to the Human Immunology Project protocol [8].

The combined detection of CD19 and CD27 markers were used to differentiate between CD19 + CD27 − naïve and CD19 + CD27+ memory B cells [5,6]. Memory B cells can be further analysed via co-staining with IgD [7] and classified into well-defined blood B cell subpopulation: SMBCs, NSMBCs, DNMBCs and NBCs (see Table 1).

Peripheral blood samples were collected in EDTA tubes and stained with fluorochrome-conjugated monoclonal antibodies against B cell markers [17,18]:

anti-CD19 PC5.5 clone J3-119, Beckman Coulter,
anti -CD27 PE-Dylight 594 clone LT27, EXBIO, Praha SA
anti-IgD FITC clone IA6-2, ThermoScientific LSG,
anti-CD45-PC7 clone J33 Beckman, Coulter,
anti-IgM PE clone SA-DA4 Beckman, Coulter.

After staining for 20 min at 4 °C, erythrocytes were lysed and cells were washed twice with PBS and resuspended in 500 µL PBS for immediate flow cytometric acquisition and analysis using a Navios EX flow cytometer (Beckman Coulter, Sykesville, MD, USA).

Doublets were excluded by plotting forward scatter height versus forward scatter area, and single cells were classified as monocytes, lymphocytes or granulocytes based on forward and side scatter characteristics. Lymphocytes are gated according to size, CD45 versus SSC dot plot centred on lymphocytes removing debris. At least 20,000 CD19+ events (total B cells) of each sample were analysed within the lymphocyte population and B cells were identified using a CD19 vs. SSC [17,18].

Absolute numbers of B cells were calculated from the patient’s WBC count and immunophenotypic data (absolute numbers of B cell subsets were based on the proportion (%) of B cells within the lymphocyte population combined with the absolute number of lymphocytes from the WBC count).

**Table 1:** Identification of memory B cell populations by means of flow cytometry.

B Cell Subset Phenotypes	B Cell Population, References
CD19+	total B lymphocytes, TBL, [18,19]
CD19+ CD27+ IgD−/+	total memory B cells, TMBCs, [19,20,21]
CD19+ CD27+ IgM+ IgD+	peripheral equivalent to marginal zone B cells, peripheral equivalent of MZBCs [22,23]
CD19+ CD27+ IgD−	class-switched memory B cells, SMBCs, [19]
CD19+ CD27+ IgD+	class non-switched memory B cells, NSMBCs, [19,24]
CD19+ CD27− IgD−	double negative (with memory properties) DNMBCs, [25,26,27]
CD19+ CD27− IgD+	naïve B cells, NBCs, gained from total memory B cells [17,18]

### 2.6. Statistics and Cluster Analysis

The distribution of quantitative variables was tested for normality using the Shapiro–Wilk test. Equality of variances was tested using Levene’s test. For quantitative variables, the results were reported as mean (standard deviation) or median (interquartile range), while for qualitative variables, they were reported as the number of patients (percentage). A non-parametric Wilcoxon signed-rank test for paired samples was used to compare the measurements obtained before the transplantation with those obtained after the transplantation. A cluster analysis was performed on the immunophenotypic profile of 47 patients utilizing the Agglomerative Hierarchical Clustering (AHC) method to establish a clustering model. This was carried out by analysing together the subpopulations of NBCs and MBCs, including TMBCs, SMBCs, NSMBCs, DNMBCs and the peripheral equivalent of MZBCs, in the transplant recipients.

After scaling all measured variables using Z-score, the immunophenotypic profiles of patients with similar distances (degree of similarity between patients in distance-based clustering algorithms) were clustered homogeneously. Agglomerative hierarchical clustering (AHC) continuously merges data vectors (in this case, patients) according to predefined distance criteria, thus creating a hierarchical clustering. In our algorithm, we selected Euclidean distance and Ward’s method as the distance metric and linkage criterion, respectively, as this combination showed optimum performance in our model. Transplant recipients were grouped based on this analysis (cluster analysis). Our final model was represented by the cluster combination with the optimal model performance, which had the highest average Silhouette score and best patient allocation.

To assess cluster differences at the subpopulation level, we used a regression approach. We applied linear regression to each of the six cell subpopulations, taking the cluster as an independent variable and controlling for potential confounders such as age, dialysis duration and type of transplant donation that could influence clustering. Our final regression models were constructed via the process of purposeful selection of variables. Variables with *p* < 0.2 were included in the final model for each combination of independent variable and outcome. We either modified or rejected the model if the regression assumptions of linearity, independence, normality and equal variance of residuals were not met.

GFR related to renal status was estimated for patients in cluster 1 and cluster 2, and cluster distributions were compared between them and with complete data representative.

Patients with similar B cell memory profile and functional status were compared with patients from the other cluster on clinical and transplant characteristics using the non-parametric Mann–Whitney U test for independent samples for quantitative variables and Fisher’s exact test of contingency tables for categorical variables. Hommel’s correction was used for multiple comparisons. Values with *p* < 0.05 were considered significant.

All parts of the statistical and cluster analysis were performed in R 4.3.

## 3. Results

### 3.1. The Effect of Transplantation on B Lymphocytes and Naïve and Memory B Cell Subpopulations

B cells are labelled with anti-CD19, -CD27 and -IgD monoclonal antibodies (mAbs) and analysed using multicolour flow cytometry. This allows for identification of five distinct B cell subpopulations: (1) NBCs (CD19+ CD27− IgD+) (2) NSMBCs, (CD19+ CD27+ IgD+); (3) SMBCs B cells (CD19+ CD27+ IgD−); (4) DNMBCs (CD19+ CD27− IgD−).

Peripheral equivalent to marginal zone B cells were also analysed (see Table 1).

To investigate any changes in the B cell compartment resulting from transplantation, we calculated the frequencies of each memory B cell subpopulation at T0 and T6 (see Table 2), and analysed the relative proportions of B cell subsets among CD19+ B cells in kidney transplant recipients at these two time points.

**Table 2:** Differences in the frequencies of B cells subpopulations in kidney transplant patients at T0 and T6.

B Cell Populations	Τ0	Τ6	*p*
Cell Subpopulations (% Cells)	*Ν* = 47 Median (IQR)	*Ν* = 47 Median (IQR)	
CD19+, total B cells	7.8 (5.9, 12.3)	6.8 (5, 9.6)	0.060
Naïve B cells, NBCs	76.4 (66.3, 84.9)	71 (59, 77.6)	0.032
Total memory B cells, TMBCs	24.6 (15.9, 37.1)	27 (20.9, 38.7)	0.280
Class-switched memory B cells, SMBCs	13.2 (9.7, 21)	15 (9.8, 19.9)	0.957
Class non-Switched memory B cells, NSMBCs	8.5 (3.8, 13)	10 (49, 16.7)	0.220
Peripheral equivalent to marginal zone B cells, peripheral equivalent to MZBCs	16.3 (4.7, 35.7)	22.8 (11.8, 44.6)	0.118
Double negative with memory properties cells, DNMBCs	12 (7.7, 18.8)	11.1 (7.6, 16.7)	0.875

**Figure 2:**
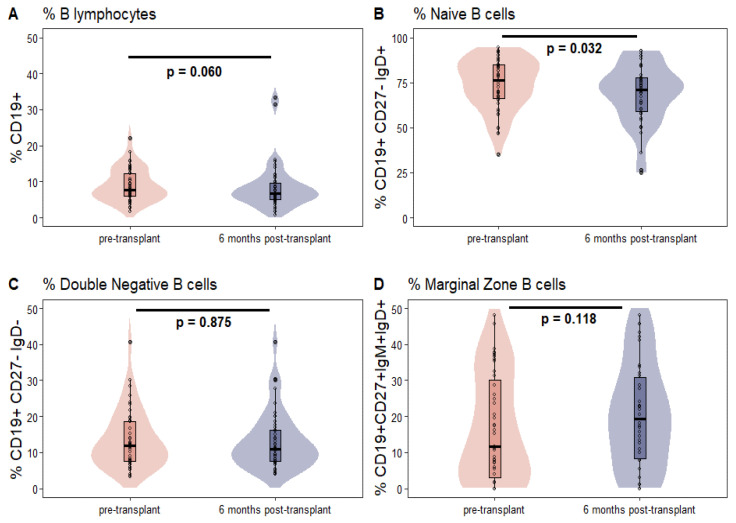

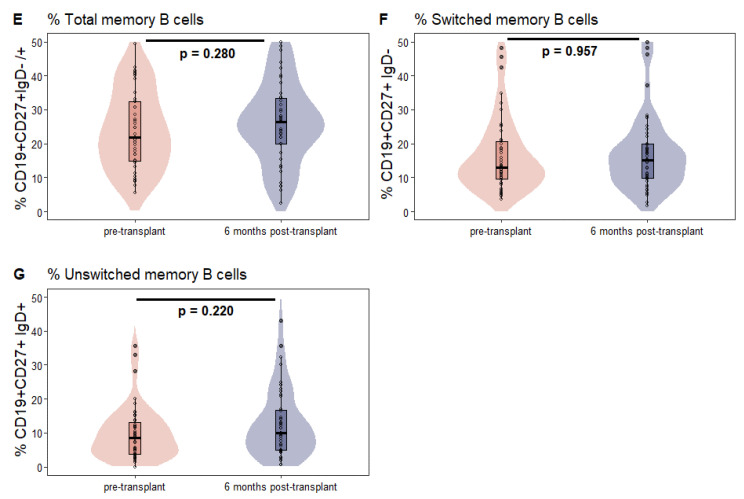
Frequencies of B cells (**A**), naïve B cells (**B**), Double negative B cells (**C**) and marginal zone B cells, peripheral equivalent (**D**) at T0 (pre-transplant) and T6 (6 months post-transplant). Frequencies of total memory B lymphocytes (**E**), class-switched (**F**), class non-switched (**G**) memory B cells at T0 (pre-transplant) and T6 (6 months post-transplant).

In general, transplantation led to a reduction in B cell frequencies at T6 as compared to T0 frequencies. However, there was no statistically significant difference observed, with a *p*-value of 0.06. A statistically significant decrease (*p*-value = 0.032) in NBCs was observed, along with a non-significant increase in the frequencies of TMBCs (*p*-value = 0.280) and their subpopulations, namely NSMBCs (*p*-value = 0.22), SMBCs (*p*-value = 0.957) and DNMBCs (*p*-value = 0.875) (see Table 2). Similar results were also observed for MZBCs, with a *p*-value of 0.118 (refer to Figure 2).

The sample population was divided according to PRA (panel reactive antibodies) into group 1 (*n* = 30) with negative PRAs and group 2 (*n* = 17) with positive PRAs. When we compared the immunophenotypes of the B cell populations studied for the two groups, there was no statistically significant difference. The same was true for HLA mismatches.

It is important to note that transplantation itself involves all the factors that determine the immune and clinical status of transplant candidates as well as the clinical course of the recipients.

### 3.2. Clustering Results

Using the Agglomerative Hierarchical Clustering algorithm, two distinct clusters of cell subpopulation proportions (proportion model) were generated, i.e., cluster 1 and cluster 2, from 47 stable renal allograft recipients 6 months after transplantation; cluster 1 consisted of 37 patients (79%) and cluster 2 of 10 patients (21%). An attempt at clustering via absolute numbers of cell subpopulations failed.

**Figure 3:**
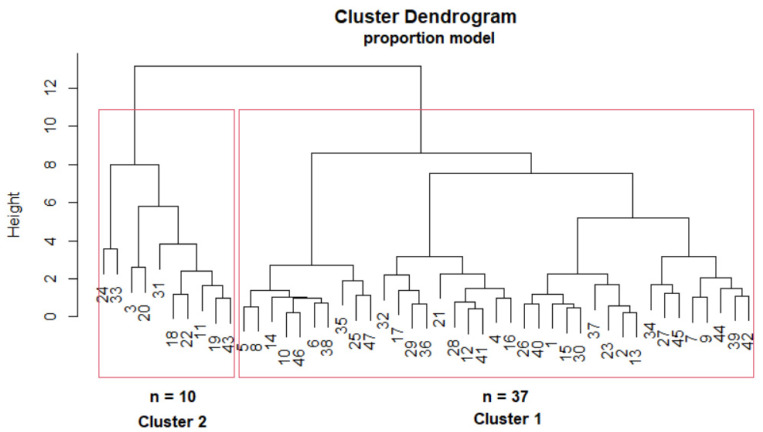
The dendrogram of the proportion model: the structure of the final hierarchy and the two clusters, represented by rectangles, that separate the 10 patients from the 37 patients (left; cluster 2, right; cluster 1).

The corresponding dendrogram of the clustering model is shown in Figure 3. The patients are shown on the *x*-axis. The height of the line connecting the patients is similar to the closeness between them; the lower the height, the greater the similarity.

**Figure 4:**
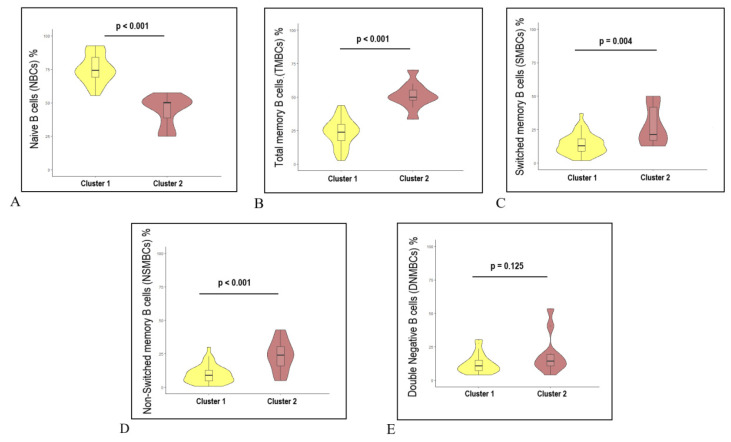
Comparisons of cell frequencies between clusters 1 and 2. (**A**). The two clusters differed in NBCs, of which cluster 1 had 74.3% and cluster 2 had 50% *p* < 0.001 (**B**). The TMBCs showed a difference between the two clusters, with cluster 1 having the lowest percentage with a median of 23.8% and cluster 2 with 49.9% *p* < 0.001. (**C**). Switched memory B cells (SBM) cells differ between the two clusters. Cluster 2 has the highest percentages (median percentage of 21.5) while cluster 1 has the lowest percentages (median percentage of 13) *p =* 0.004. (**D**). Non-switched memory B cells (NSBM) are different in cluster 1 and cluster 2, with the median percentage of 9 in cluster 1 and a median of 24 in cluster 2 (*p* < 0.001). (**E**). DN cells do not reach significant different levels between the two clusters (*p* = 0.125).

Table 3 and Figure 4 show the median percentages (IQR), along with the *p*-values, for each subpopulation in clusters 1 and 2.

**Figure 5:**
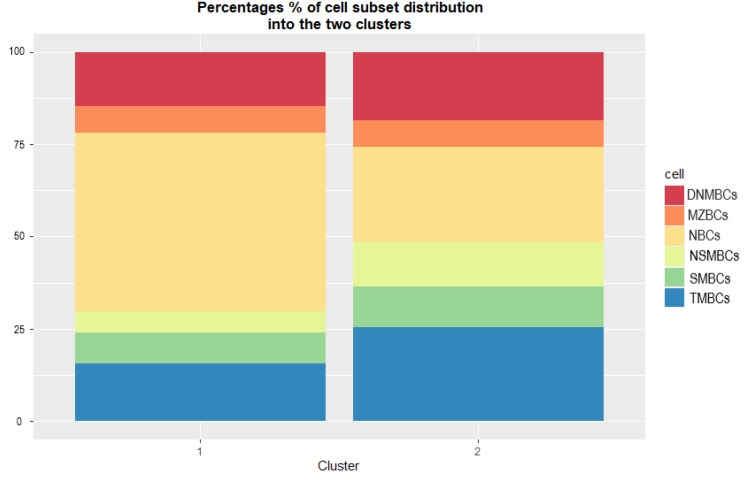
Bar plots showing the distribution of cells in each cluster. The length of each rectangle is calculated as the proportion of the total score of that variable in the cluster. Double negative (with memory properties) cells: DNMBCs, peripheral equivalent to marginal zone B cells: MZBCs, naïve B cells: NBCs, class non-switched memory B cells: NSMBCs, class-switched memory B cells: SMBCs, total memory B cells: TMBCs.

In addition, to illustrate the contribution of each variable in each cluster, the median proportions of the cell subpopulation in the two clusters were plotted using bar plots (see Figure 5). Note that this distribution is not a normal distribution.

**Figure 6:**
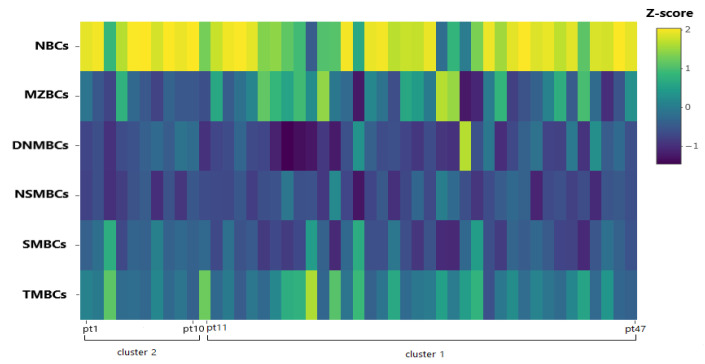
Heatmap of patient distribution at intra-cluster level. The heatmap matrix is constructed by all B cell subpopulations of our proportion model in its rows and the renal transplant recipients in its columns. Z-scaled scores of all the variables for each patient in our model are represented by colour grading. The first ten observations are the 10 patients of cluster 2 (patient 1 to patient 10), while the remaining 37 patients belong to cluster 1 (patient 11 to patient 47). Naïve B cells: NBCs, peripheral equivalent to marginal zone B cells: MZBCs, double negative with memory properties cells: DNMBCs, class non-switched memory B cells: NSMBCs, class-switched memory B cells: SMBCs, total memory B cells: TMBCs, pt: patient.

Finally, a heat map of the Z-score scaled patient scores of the subsets was generated to compare the intra-cluster distribution of variables among the patients. The heat map in Figure 6 clearly shows that cluster 2 has a more memory-dominated phenotype with lower proportions of NBCs, whereas cluster 1 has patients with high proportions of NBCs and lower scores for the memory subpopulations.

### 3.3. Proportion Model Cluster Distinction

Linear regression modelling was used to explore cell subpopulation differences between clusters and also account for factors that might influence the association of variables between clusters in our proportion model. Linear regression models, one for each combination of the subpopulations of cells (dependent variable) and independent variables were constructed. Clusters were used as the main independent variable in order to find inter-cluster differences (factor variable with levels ‘one’ or ‘two’), while variables such as age (numerical variable), donor type (factor variable with two levels; ‘deceased’ or ‘living’ donor) and dialysis duration (numerical variable) represented the covariates of our models. Univariate models were firstly constructed and then their independent variables were selected to build the final multivariable or univariate models for the six subpopulations dependent variables. The linear regression assumptions were not violated in every case so no adjustment was needed.

NSMBC, DNMBC and MZBC models were univariate and had only ‘cluster’ as an independent variable.

TMBC and SMBC had ‘age’ and ‘donor type’ as additional explanatory variables and NBC had ‘age’ to improve the models. All models except that for MZBC cells suggested that the two clusters were well defined, with a statistically significant difference between them for the cell subpopulations studied (*p* < 0.05 in the variable ‘clusters’, Table 4). Linear regression demonstrated a difference in the cell subpopulation composition between clusters when factors such as age, donor type, and duration of dialysis were analysed, verifying previous clustering results, except for one subpopulation.

### 3.4. Study Patients

The main characteristics of the 47 renal allograft recipients are summarized in Table 5.

Both cluster 1 and cluster 2 had a higher proportion of males, the former with 71% and the latter with 70% (Table 5). The mean age was 49.6 years in cluster 1 and 52.5 years in cluster 2. The mean duration of dialysis was 94.8 months in cluster 1 and 82 months in cluster 2. Cold ischaemia time was longer in cluster 1 (19.2 h) than in cluster 2 (15.9 h), but the differences were not significant. In both clusters, the majority of patients had previously been on haemodialysis (HD), only one patient in each cluster had been on continuous ambulatory peritoneal dialysis (CAPD), and three patients in cluster 1 and one patient in cluster 2 had been on both types of dialysis at different times. Delayed graft function, diabetes and hypertension were almost equally distributed in each cluster, with similar percentages. In cluster 1, deceased donor grafts accounted for 78% and living donor grafts for 22%, whereas in cluster 2, the first category accounted for 60% and the second for 40%, Table 5.

We compared the median absolute counts or percentages of cells between the two clusters of variables that were not directly included in our model (see Table 3). We examined the differences in absolute cell counts of the cell subpopulations within the proportion model between the clusters. Only one subpopulation exhibited a noteworthy difference in absolute counts. The absolute counts of NBCs showed a statistically significant difference (*p* = 0.002), with cluster 1 having the highest count of 62.3 compared to only 27.8 in cluster 2. The absolute counts of TMBCs showed no statistically significant difference between cluster 1 and cluster 2. We also evaluated total B cells. Table 5 shows that there was no significant difference in the total number of B cells between cluster 1 (81) and cluster 2 (78). The percentage of total B cells was not significantly different either.

#### 3.4.1. eGFR Distribution of the Two Clusters 1 and 2

**Figure 7:**
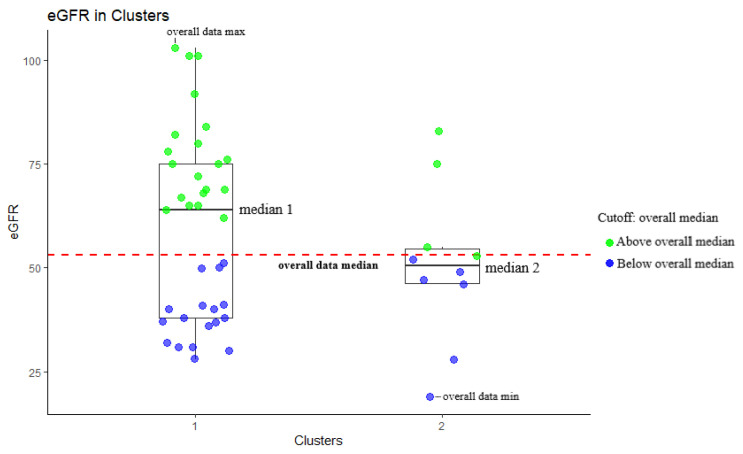
Boxplots of eGFR of the patients in clusters with median values of each cluster (median 1, median 2). Overall data median was also estimated and depicted with the horizontal intermittent red line (overall data median, cutoff). Overall data maximum belongs to cluster 1 (overall data max) while overall data minimum belongs to cluster 2 (overall data min). Patients are shown as points. Green points represent patients with eGFR value above or equal with overall median and blue points represent patients with eGFR value below overall median.

We analysed the eGFR distribution for both clusters. Each cluster was plotted as a boxplot. The points represent the patients’ eGFR (see Figure 7). The boxplot shows the median values for each cluster, and a horizontal intermittent red line depicts the overall median eGFR. The median eGFR of all patients was 53, with an interquartile range (IQR) of 39–75. The median (IQR) eGFR of cluster 1 was 64 (38, 75), and that of cluster 2 was 50.5 (46.25, 54.5).

In cluster 1, 20 out of 37 patients (54%) had an eGFR higher than the overall data median. Moreover, the median eGFR of this cluster exceeded the overall median, indicating a trend of higher median eGFR for this cluster. In cluster 2, three values were above the overall median, one had the same value, while six out of ten values (60%) and the cluster’s median were below the overall median. In cluster 2, eight out of ten patients (80%) also had eGFR values under 60. The maximum and minimum values in the overall dataset were in cluster 1 and cluster 2, respectively.

In conclusion, patients in cluster 1 typically exhibited higher values of eGFR, while in cluster 2, most patients had eGFR values below 60. Although the statistical comparison was not significant (*p* = 0.405, see Table 5), the different distribution of the two clusters was evident when compared. The small sample size of the study may have contributed to the lack of statistical significance.

#### 3.4.2. The Effect of Donor Type, Age of Recipient, Dialysis Duration on B Cells, Naïve and Memory B Cell Subpopulations

A critical clinical question is whether the donor type affected the allograft function in our study, especially in B cell subpopulations.

Two populations, one with a low risk of immunogenicity—living donors (most with negative PRA, low number of HLA mismatches and short duration of dialysis)—and the other with a high risk of immunogenicity—deceased donors (usually with positive PRA, high number of HLA mismatches and long duration of dialysis) were considered.

Out of a total of 37 donors in cluster 1, 8/37 (22%) were living donors, and the remaining 29/37 (78%) were deceased donors. In cluster 2, 40% of the patients had living donors while 60% had deceased donors, and this distribution was dominant in both clusters. However, no statistically significant difference was found between the two clusters in terms of donation type (*p* = 0.251). The type of donor did not affect the different phenotypes of the B-subpopulation. Age and dialysis duration did not differ significantly between cluster 1 and 2, which is consistent with the results regarding the type of donor.

## 4. Discussion

Detailed knowledge of the distribution of B cell subpopulation in the peripheral blood reflects the immune status of an individual and may also contribute to a better understanding of B cell involvement in transplantation [28]. There is a large body of literature on T cell and solid organ transplantation, but B cells have not received the attention they deserve and the published results present a mixed picture.

A cohort study was conducted to investigate changes in the distribution of peripheral blood B cell subpopulations in kidney transplant recipients 6 months after transplantation. The study also aimed to explore any association between naïve and memory B cell subpopulations and renal function at this time point. Previous studies have classified graft acceptance or rejection based on an examination of naïve and memory B cells, or the ratio of naïve-to-memory B cells [29,30].

We found changes in the immunophenotypes of naïve and memory B cell subpopulations at T6 compared to T0 values. There was a significant decrease in the frequency of naïve B cells and a trend towards a lower percentage of total B cells 6 months after transplantation, which could be explained by the decrease in naïve cells (which are in the highest proportion in the B cell compartment). It is not clear whether the decrease in naïve B cells after transplantation is due to depletion of existing cells or to a low rate of cell synthesis in the bone marrow. A concomitant non-significant increase in total memory B cells was seen, probably due to a non-significant increase in both switched and non-switched B cells.

We agree with the study by van de Berg, P.J.E.J. and colleagues [31], who also found a more differentiated B cell profile, but at different time points and the values were compared to those of healthy individuals. Furthermore, they found no difference in B cell populations between recipients with stable graft function and those with rejection and agreed with us that there were no significant changes in marginal zone B cells, anticipating Alfaro R et al. 2021 [32], who found a decrease after transplantation. Another group [15] also found a lower percentage of MZB, but in kidney transplants diagnosed with rejection compared to the rejection-free group. Zhuang et al. [33] found reduced B cells in comparison to healthy controls, but similar to our findings. They also found decreased MZB in comparison to ESRD patients and healthy controls. Regarding memory subpopulations, they observed no difference one and five years after transplantation.

The immunosuppression regimen was the same for the enrolled patients, and the induction therapy did not have an effect on the populations studied. All patients had stable graft function and did not report any viral or bacterial infections. It is known that infections can increase memory B cells. Out of the patients, only one suffered rejection, but was not included in the study.

In line with our findings, Schuller and colleagues [34] also noted a decrease in the frequency and absolute number of B cells. However, this was at a different time point than our study. They differed from us in the observation of naïve B cells, where there was no change in frequency or absolute number one year after transplantation. In their study, they also observed a significant increase in DN, but at T6 we did not observe any change, which might be attributed to the smaller sample size and different time point employed in our study. There is a general lack of data on the DN population in transplantation.

Regarding memory cells, their study reported that class-switched memory B cells tended to be at lower frequencies, whereas non-switched memory B cells remained stable in patients one year after transplantation, which is different from our results at T6. Our findings were consistent with those of Wang L et al. [35], although they evaluated B cell subpopulations three years after transplantation. They identified a significant reduction in the absolute numbers of naïve B cells and a significant increase in memory B cells. Most of the B cell subpopulations demonstrated no significant difference, except for the occurrence of a higher frequency of switched memory B cells and reduced frequency of non-switched memory B cells in transplanted patients compared to those in the healthy control group, but not compared to the pre-transplantation values.

Unlike our findings, Svadova and colleagues [36] did not report any alteration in the naïve cells. Their approach involved applying different immunomarkers to phenotype cells at different time points. They observed an increase in frequency and absolute numbers of memory cells and total B cells in the first week, but this was followed by a decline at three months after transplantation, which persisted for one year. Alfaro et al. [32] obtained similar results to ours, reporting a decrease in total and naïve B cells six months after transplantation, and an increase in all memory subpopulations.

Validating results of B lymphocyte subpopulations across different studies is difficult because they are conducted at varying study points after transplantation among renal allograft patients. Additionally, different surface markers are employed to define the relevant populations by phenotype, resulting in uncertainty regarding the targeted subpopulation.

To examine whether there is a restoration of cell populations after transplantation, our study utilizes pre-transplant values from the recipients rather than from healthy individuals, as transplant candidates often display phenotypic and functional changes in peripheral lymphocytes [33,37]. To strengthen our control selection, previous studies have shown reductions in the proportion of naïve B cells [38], as well as memory and regulatory B cells in uremic patients [33].

Using unsupervised machine learning clustering and Agglomerative Hierarchical Clustering algorithm, transplant recipients were grouped based on their immunophenotypic profiles of naïve and memory B cell subpopulations, six months after transplantation. Two distinct patient clusters, namely cluster 1 and cluster 2, with cell subpopulation frequencies (proportional model), were identified.

When conducting a study, it is vital to determine whether to evaluate the frequencies of immunophenotypes and/or the absolute numbers of various populations [39,40,41]. Although unsuccessful, it is noteworthy that attempts were made to perform cluster analysis using absolute counts. This may represent a limitation of the study.

We investigated how the profile of naïve and memory B cell subpopulations in both identified clusters relates to eGFR, which measures renal function. Renal function is critical as it has been reported in literature that reduced eGFR one year after transplantation can cause graft failure [42,43].

The characteristics of cluster 1 include a high frequency and absolute number of naïve cells, as well as a low frequency and absolute number of total memory cells. Conversely, cluster 2 is characterized by a low frequency and absolute number of naïve cells, alongside a high frequency and absolute number of memory cells. Concerning eGFRs, it was discovered that individuals in cluster 1 had a median eGFR of 64 mL/min/1.73 m^2^, while patients in cluster 2 presented a lower eGFR value of 50 mL/min/1.73 m^2^. High frequencies and absolute numbers of naïve B cell populations are associated with improved renal function, an observation also made in patients belonging to cluster 1. In relation to the memory subpopulations, both class-switched and nonswitched, their frequencies and absolute numbers are significantly higher in cluster 2, which accounts for the elevated levels of memory cells. The increase in the number of memory B cells is linked to allospecific responses, which may be associated with antibody production. There was no difference in DN in both clusters.

Multivariable linear regression analyses in clusters 1 and 2 showed that the results were independent of age, type of donation and duration of dialysis. This contrasts with the findings of Gama and colleagues [39], who reported a significant association between age and the absolute numbers of B cells but not with their frequencies.

The patient data analysed from clusters 1 and 2 differ from those obtained from the overall study population, demonstrating that our unsupervised machine learning analysis provides a new stratification of transplant patients six months after transplantation based on naïve and memory B cell immunophenotyping, highlighting the importance of these cell subpopulations and linking them to renal function via eGFR.

There is ample evidence to suggest that circulating memory B cells play a central role in an episode of AMR, particularly in sensitised recipients or paediatric patients [44]. In transplanted patients with antibody-mediated rejection, the ratio of naïve to memory cells was decreased [30].

Patients in cluster 2 demonstrate a high frequency and absolute number of memory cells, as well as a low frequency and number of naïve cells, with a lower eGFR. This suggests they may lack a protective phenotype against rejection.

To verify our results regarding the protective phenotype against rejection in cluster 1, we are continuing the analysis over time.

It is important to acknowledge the limitations of this study.

First, by focusing on results from a single centre, with a relatively small sample size (*n* = 47), with two study time points, one pre-transplant and the other six months post-transplant, and using pre-transplant values as controls rather than those of healthy individuals, the results would be biased. For this reason, the ongoing research study will also include healthy control subjects and additional later time points to provide information on long-term changes. 

Second, the results of the study may have been confounded by the limitation of using single centre sample population consisting of a non-diverse group of stable transplant recipients on a uniform immunosuppressive regimen, without a group of patients with episodes of rejection, and with limited evaluation of transplant recipients with donor-specific antibodies. In this case, it will be important to address these limitations in future research and the results of the studies could be validated in larger cohorts.

Finally, a limitation of our study is that the clustering analysis performed only on the frequency of the B cell memory phenotype failed in absolute numbers, and the eGFR values in the resulting clusters did not show a statistically significant difference. This is probably due to the small sample size coming from a single centre, and this limitation could be addressed by participation in multicentre trials, which would also allow for sample diversity and possible application of different immunosuppressive protocols to consider any effect on B cell subpopulation.

Furthermore, interventional studies could determine whether changes in B cell phenotypes have an impact on transplantation outcomes.

In conclusion our short-term study suggests that there is B cell heterogeneity in kidney recipients with stable graft function.

Unsupervised machine learning can define clusters based on the immunophenotypes of the memory sub-populations and the naïve B cells, which leads to an improved stratification of patients in relation to their eGFR.

## Figures and Tables

**Table 3:** Median (IQR) percentages of cell subset proportions in cluster 1 and cluster 2. Total memory B cells: TMBCs, class-switched memory B cells: SMBCs, class non-switched memory B cells: NSMBCs, peripheral equivalent to marginal zone B cells: MZBCs, double negative with memory properties cells: DNMBCs, naïve B cells: NBCs.

Cell Subpopulation (% Cells)	Cluster 1, *Ν* = 37 Median (IQR)	Cluster 2, *Ν* = 10 Median (IQR)	*p* Value
Total MBC	23.8 (17.3, 9.6)	49.9 (47.7, 55)	<0.001
SMBC	13 (9, 8)	21.5 (16.8, 41.6)	0.004
NSMBC	9 (4.7, 2.8)	24 (16, 30.3)	<0.001
DN MBC	10.8 (7.2, 5)	14.3 (10.8, 19.4)	0.125
NBC	74.3 (69.2, 4)	50 (38.7, 50.5)	<0.001
MZBCs	22.7 (9.9, 2)	36.3 (17.5, 47.4)	0.13

**Table 4:** Linear regression models between the two clusters formed. Each row of the table represents a single model for each cell subpopulation. The adjusted model column lists the variables of each model and the remaining columns show the estimates, *p*-values, 95% confidence intervals (CIs) and adjusted R-squared values (adjusted R^2^) of the models. Bold *p*-values indicate statistical significance (*p* < 0.05).

Subpopulation and Model	Independent variables in the model	Estimate	*p* Value	CI 95%	Adjusted R-Squared (Adjusted R^2^)
Naïve B cells- NBCs model	***age*** ***clusters***	−0.16 −30.03	0.185 **<0.001**	( −0.42, 0.08) (−37.58, −22.50)	0.59
Total memory B cells- TMBCs model	***age *** ***donor type*** ***clusters***	0.21 4.31 26.16	0.096 0.236 **<0.001**	(−0.04, 0.46) (−2.91, 11.54) (18.82, 33.49)	0.57
Class-switched memory B cells- SMBCs model	***age *** ***donor type *** ***clusters***	0.24 5.35 11.93	**0.045** 0.118 **0.001**	(0.005, 0.47) (−1.42, 12.12) (5.06, 18.80)	0.29
Class non-switched memory B cells- NSMBCs model	***clusters***	13.79	**<0.001**	(8.07, 19.51)	0.33
Double negative with memory properties- DNMBCs model	***clusters***	7.35	**0.033**	(0.61, 14.09)	0.08
Peripheral equivalent to marginal zone B cells- MZBCs model	***clusters***	8.68	0.282	(−7.39, 24.76)	0.01

**Table 5:** Baseline data for the different clusters with their *p* values. Continuous: mean (SD) or median (IQR), categorical: *n* (percentages %). TMBCs: total memory B cells, NBCs: naïve B cells.

	Study Sample *N* = 47	Cluster 1 *N* = 37	Cluster 2 *N* = 10	*p* Value Cluster1 vs Cluster2
**Sex** Female, n (%) Male n (%)	14 (30) 33 (70)	11 (29) 26 (71)	3 (30) 7 (70)	1
**Age in years, mean (SD)**	51(12)	49.6 (11)	52.5 (16)	0.52
**Type of donor,** n (%) Deceased Living	35 (74) 12 (26)	29 (78) 8 (22)	6 (60) 4 (40)	0.251
**Duration of dialysis(months), mean (SD)**	89 (47)	94.8 (44)	82 (49)	0.442
**Type of dialysis** n (%) HD CAPD CAPD + HD	41 (87) 2 (4) 4 (9)	33 (89%) 1 (3%) 3 (8%)	8 (80%) 1 (10%) 1 (10%)	1
Delayed graft function		Yes: 12 (32%) No: 25 (68%)	Yes: 3 (30%) No: 7 (70%)	1
Cold ischaemia time (hours)), mean (SD)		19.2(4.6)	15.9(4.7)	0.053
Hypertension, n (%) Yes No		25 (67) 12 (33)	6 (60) 4 (40)	0.716
Diabetes, n (%) Yes No		4 (11) 33 (89)	1 (10) 9 (90)	1
**Distribution of underlying kidney disease**				
Polycystic kidney disease%	21%			
Primary glomerulopathies%	21.5%			
Reflux nephropathy%	13%			
Diabetes mellitus%	4%			
Nephrosclerosis/hypertension%	4%			
Urinary tract infections/stones%	3%			
Other%	18%			
Unknown%	15.5%			
Induction therapy:				
Basiliximab, n (%)	40 (85)	30/37patients	7/10patients	
ATG, n (%)	7 (15)	7/37patients	3/10patients	
Maintenance immune suppression:				
tacrolimus/mycophenolate/prednisone%	100.0%			
eGFR (mL/min/1.73 m^2^), median (IQR)	53 (39–75)	64 (38, 75)	50.5 (46.25, 54.5)	0.405
**Cell subpopulations, median (IQR)**				
B cells—absolute		81 (49.2, 142.4)	78 (54.6, 123.7)	0.636
B cells%		7.5 (4.9, 10)	6.55 (5.85, 6.8)	0.475
TMBCs—absolute NBCs—absolute		17.8 (10.5, 33.8) 62.3 (33.9, 128.4)	39.2 (26.8, 59.7) 27.8 (19.2, 41.6)	0.07 0.002

## Data Availability

Upon request, the corresponding author can provide the data sets used and analysed in this study.
